# B-Site Ru Doping in Sr-Substituted LaCoO_3_ Perovskite for Enhanced OER Performance: A Combined Experimental and DFT Study

**DOI:** 10.3390/ma19112383

**Published:** 2026-06-03

**Authors:** Lina Zhang, Tian Fang, Changhai Liu, Wenchang Wang, Shiying Wang, Zhidong Chen

**Affiliations:** School of Materials Science and Engineering, Changzhou University, Changzhou 213164, China

**Keywords:** perovskite oxides, A/B co-modulation, oxygen evolution reaction, DFT calculations

## Abstract

The sluggish kinetics of the OER in alkaline media demand efficient non-precious electrocatalysts. This study develops a strategy of B-site Ru doping in Sr-substituted LaCoO_3_ perovskite to engineer its electronic structure, thereby activating the LOM. DFT calculations and electrochemical measurements were employed to investigate the electronic structure and catalytic performance. The synthesized La_0.6_Sr_0.4_Co_0.9_Ru_0.1_O_3_ (LSCR-0.1) catalyst achieves a low overpotential of 256.8 mV at 10 mA cm^−2^ and a Tafel slope of 84.88 mV dec^−1^, along with excellent long-term stability in alkaline electrolyte. The results show that Ru doping shifts the O 2p-band center and lowers the oxygen vacancy formation energy, significantly reducing the energy barrier of the rate-determining step. This work concludes that B-site doping on A-site substituted perovskites offers a general and effective strategy for modulating their electronic structure to enhance OER performance.

## 1. Introduction

Escalating energy crises and environmental degradation have intensified the search for advanced energy solutions. Hydrogen, a plentiful and environmentally benign renewable, is pivotal for sustainable development. Its production, however, remains a critical challenge due to the scarcity of free hydrogen, demanding efficient and sustainable technological advancements [1,2,3,4,5]. Water electrolysis produces high-purity hydrogen and oxygen. Notably, the oxygen evolution reaction (OER), with its sluggish four-electron transfer kinetics, becomes the key bottleneck limiting the overall efficiency of alkaline water electrolysis. Although noble metal oxides such as IrO_2_ and RuO_2_ exhibit excellent OER activity, their high cost and scarcity restrict large-scale application. Thus, developing high-performance, non-precious metal OER electrocatalysts is essential for advancing industrial-scale water electrolysis [6,7,8]. Perovskite oxides are particularly promising due to their tunable electronic structures and lattice oxygen redox mechanisms, offering a pathway toward efficient and durable OER catalysis [9,10].

Perovskite oxides (ABO_3_) possess tunable structures and oxygen vacancies. These unique properties make them top candidates for alkaline OER [9]. In its crystal structure, the A-site is commonly occupied by rare earth elements (e.g., lanthanum, praseodymium), alkaline earth metals (e.g., Ca, Sr, Ba), or alkali metals (e.g., K, Na, Rb) [11,12,13,14]. A-site cations coordinate with surrounding oxygen ions to form a twelve-coordinated cuboctahedral geometry. Meanwhile, the B-site is mainly populated by transition metals (e.g., Co, Fe, Ni) or noble metals (e.g., Ru, Pt, Ir). B-site cations occupy the center of oxygen octahedra. Bonding with six oxygen ions, they form a stable BO_6_ octahedral framework. This framework constitutes the crystal backbone and, more critically, acts as the key unit governing OER activity [14,15,16]. While A-site cations do not directly participate in redox reactions, they act as “remote regulators” that fine-tune the local environment and electronic structure of BO_6_ octahedra via charge balance, lattice distortion, oxygen vacancies, and electron transport. Hence, A-site tuning is a key strategy for boosting electrocatalytic performance [17,18,19]. B-site doping offers a more direct and effective route by reconstructing the BO_6_ coordination environment, altering crystal field splitting, and tuning the electronic configuration of central metal ions. This micro-engineering enhances intrinsic activity, increases exposed active sites, and lowers rate-determining step barriers, making B-site modification central to overcoming performance bottlenecks [20]. Recent studies emphasize that high overpotential and poor stability must be addressed. Approaches like A/B co-modulation, lattice strain engineering, and interface heterostructure design all aim to modulate electronic spin states and octahedral geometric distortion, thereby optimizing reaction kinetics [21,22]. LaCoO_3_ is cost-effective with superior redox reversibility, making it a strong candidate to replace noble metal OER catalysts. However, low surface defect density and poor interfacial charge transport limit its practical performance below theoretical values. Thus, structural modification is essential to unlock its full catalytic potential [23].

Scientists have developed a systematic strategy to optimize the OER performance of LaCoO_3_, ranging from single-site A or B substitution to dual-site modulation [24,25]. Unlike single-site doping, A/B-site synergy provides no simple additive effect but a strong synergistic effect via coupled lattice strain and charge compensation [24,25,26]. This design philosophy has been validated in various high-performance, complex-composition systems. For instance, in La_0.6_Sr_0.4_Fe_x_Co_1−x_O_3_ [27], A-site Sr doping and B-site Fe/Co regulation synergistically optimize the electronic structure and defect chemistry. Analogous enhancements in OER activity, driven by similar synergistic mechanisms, have been observed in La_0.6_Ca_0.4_Co_1−x_Mn_x_O_3_ and A/B co-modulation LaNiO_3_-based perovskites [21,28]. A/B-site synergy typically exceeds the performance limits of single-doped catalysts and greatly boosts catalytic activity.

This study combines density functional theory (DFT) calculations and experiments to investigate how B-site Ru doping on A-site Sr-substituted perovskite oxides regulates their OER performance. The literature reports suggest that La_1−y_Sr_y_CoO_3_ exhibits peak OER activity in the range y = 0.3–0.5, with y = 0.4 being the most widely adopted Sr doping level. Therefore, we chose y = 0.4 as the fixed Sr content [29,30,31]. A series of La_0.6_Sr_0.4_Co_1−x_Ru_x_O_3_ catalysts were synthesized and denoted as LSC (x = 0, La_0.6_Sr_0.4_CoO_3_), LSCR-0.05 (x = 0.05, La_0.6_Sr_0.4_Co_0.95_Ru_0.05_O_3_), LSCR-0.1 (x = 0.1, La_0.6_Sr_0.4_Co_0.9_Ru_0.1_O_3_), and LSCR-0.15 (x = 0.15, La_0.6_Sr_0.4_Co_0.85_Ru_0.15_O_3_). Among them, the LSCR-0.1 sample exhibited the optimal OER activity. Consequently, this study confirms that the combination of A-site Sr substitution and B-site Ru doping enhances OER performance by synergistically regulating the lattice structure and electronic states. It provides a theoretical foundation and a regulation pathway for developing efficient and stable non-noble metal electrocatalysts.

## 2. Experimental Section

### 2.1. Materials and Synthesis

A sol–gel approach was employed to fabricate the Sr-doped and Ru-doped LaCoO_3_ perovskite oxides. The general formula is La_0.6_Sr_0.4_Co_1−x_Ru_x_O_3_, denoted as LSCR-x (x = 0, 0.05, 0.1, 0.15). Co(NO_3_)_2_ · 6H_2_O, and RuCl_3_ with a total molar amount of 5 mmol, where the molar ratio of Co to Ru is (1−x):x, La(NO_3_)_3_ ·6H_2_O (3 mmol), and Sr(NO_3_)_2_ (2 mmol) were dissolved in 50 mL of deionized water. Citric acid (12 mmol) and EDTA (8 mmol) were then added sequentially under stirring. The pH was adjusted to 8–9 using ammonia solution. The sol–gel transition was driven by heating the mixture at 90 °C under stirring, yielding a gel that was dried at 200 °C for 6 h. To promote phase formation, the ground precursor was calcined at 750 °C for 6 h with a controlled heating rate of 5 °C min^−1^ and finally ground into powder for further use.

### 2.2. Physicochemical Characterization

X-ray Diffraction (XRD): Crystal structures of the materials were characterized using a Rigaku D-MAX2500 XRD (Rigaku, Tokyo, Japan) (Cu Kα radiation, λ = 1.54056 Å). Characterization was executed with the X-ray tube powered at 40 kV and 40 mA, covering a 2θ window of 5–80°.

X-ray Photoelectron Spectroscopy (XPS): Surface elemental composition, chemical states, and valence distributions were analyzed qualitatively and quantitatively using a Thermo Fisher Escalab 250Xi XPS system (Waltham, MA, USA).

Scanning Electron Microscopy (SEM): Particle sizes and morphological features were examined using a JEOL JSM-6360LA (JEOL, Tokyo, Japan) and a Zeiss SUPRA-55 field-emission scanning electron microscope (Zeiss, Oberkochen, Germany).

DFT calculations: Vienna Ab initio Simulation Package (VASP, version number-VASP 6.3, Vienna, Austria) was adopted to carry out DFT calculations. The Perdew–Burke–Ernzerhof (PBE) functional was selected for the calculations, and the DFT-D3 method was introduced to make corrections for van der Waals interactions. Considering the strong correlation characteristics of d-electrons in transition metals, Hubbard U values of 4.0 eV and 3.0 eV were respectively applied to Co and Ru elements.

### 2.3. Electrochemical Tests

Electrochemical tests were conducted on a CHI760E workstation (CH Instruments, Shanghai, China), with electrochemical impedance spectroscopy (EIS) measured on a Parstat 3000 (Princeton Applied Research, Oak Ridge, TN, USA). Catalyst ink was prepared via dispersion of 1.5 mg of catalyst and 1.5 mg XC-72 carbon black in 180 μL H_2_O and 60 μL ethanol, followed by 10 μL Nafion (0.5 wt%). After 80 min ultrasonication, 3 μL of the ink was drop-cast onto a 3 mm GCE (loading: 0.326 mg cm^−2^).

OER evaluation was conducted in 1.0 M KOH with a three-electrode configuration (Hg/HgO reference, graphite rod counter electrode). Potentials were converted to RHE via: *E*_RHE_ = *E_Hg/HgO_* + 0.059 pH + 0.098V [26].

Linear sweep voltammetry (LSV) was employed to record the polarization profiles for the OER. Based on the LSV data, Tafel plots (*η* = *b* log *j* + a) were derived via curve fitting, where the parameter b represents the Tafel slope. Electrochemical double-layer capacitance (*C_dl_*) was determined from cyclic voltammetry (CV) scans at 20–120 mV s^−1^ (step: 20 mV s^−1^). The electrochemical active surface area (ECSA) was calculated as ECSA = *C_dl_/C_s_* (*C_s_* = 40 μF cm^−2^) [26,32].

EIS scans (100 kHz–0.1 Hz) were executed with a 5 mV AC perturbation [26].

Stability was assessed by the following: (i) accelerated degradation test (1000 CV cycles at 100 mV s^−1^) and (ii) 10 h chronopotentiometry at 10 mA cm^−2^.

## 3. Results and Discussions

### 3.1. Characterization of Catalyst Structure and Morphology

**Figure 1:**
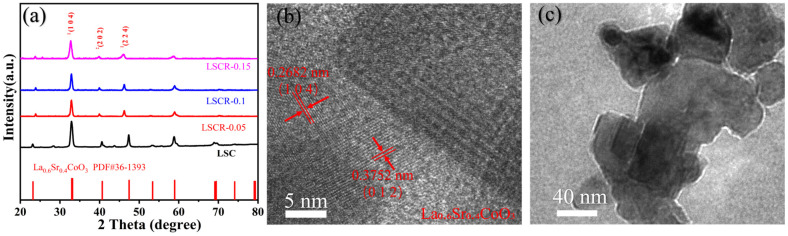
(**a**) XRD patterns of LSC, LSCR-0.05, LSCR-0.1, and LSCR-0.15. Lattice parameters obtained from Rietveld refinement are summarized in Table S3. A HRTEM (**b**) and TEM (**c**) image of LSCR-0.1.

To verify the successful doping of Ru and Sr into LaCoO_3_(LSC), we characterized the samples by XRD. The results are shown in Figure 1a. The diffraction peaks of LSC, LSCR-0.05, LSCR-0.1, and LSCR-0.15 correspond well to the standard patterns of La_0.6_Sr_0.4_CoO_3_ (JCPDS No. 36-1393), respectively. This confirms that all samples possess the desired perovskite crystal structure without obvious impurity phases, providing a solid structural basis for the successful incorporation of dopant elements. For the doped LSCR samples, the main diffraction peaks at 33.178°, 40.681°, 47.462°, and 58.969° can be indexed to the (104), (202), and (024) crystal planes of La_0.6_Sr_0.4_CoO_3_ according to the PDF standard cards. The precise matching of the above characteristic diffraction peaks confirms that Ru and Sr have been successfully doped into the LSC perovskite lattice.

The XRD patterns of samples with different Ru contents in Figure 1a further confirm the doping effect. At a low Ru doping level (LSCR-0.05), no obvious impurity peaks appear. This indicates that low Ru doping does not destroy the perovskite structure of LSC and Ru is uniformly dissolved into the lattice. As the Ru doping level increased to LSCR-0.1 and LSCR-0.15, the characteristic diffraction peaks of the samples shifted to lower angles, indicating lattice expansion. The lower-angle shift in the XRD peaks indicates lattice expansion upon Ru doping. XPS analysis confirms that Ru mainly exists as Ru^4+^ rather than Ru^3+^. Since both Ru^4+^ (ca. 62 pm) and Ru^3+^ (ca. 68 pm) have larger ionic radii than Co^3+^ (ca. 61 pm), the observed peak shift is consistent with Ru substitution. Furthermore, the presence of Ru^4+^ enhances metal–oxygen covalency and significantly intensifies the distortion of CoO_6_ octahedra. Given that the perovskite framework is highly sensitive to variations in ionic radius, dopants with mismatched sizes often induce lattice distortions. The doping of Ru promotes CoO_6_ octahedral distortion precisely through this mechanism, which is critical for activating the lattice oxygen mechanism [33]. This distortion effect subsequently promotes an increase in the oxygen vacancy content within the perovskite lattice. This drives the O 2p-Co 3d orbital hybridization approaching the Fermi level, directly underpinning the subsequent improvement in electrocatalytic performance.

Scherrer analysis based on the perovskite (104) diffraction peak yields crystallite sizes of 15–20 nm (Table S2), and the sharp diffraction peaks confirm the good crystallinity of all samples. Rietveld refinement was performed on the XRD patterns using the rhombohedral space group R-3c. The refined lattice parameters (a, c, and unit cell volume) as well as the goodness-of-fit indicators (R_wp_ and GOF) are summarized in Table S3. The low R_wp_ values (all below 5.5%) and GOF values close to one indicate good fitting quality. With increasing Ru content, both a and c lattice parameters increase monotonically (e.g., for LSCR-0.1, a = 5.44546 Å and c = 13.20932 Å), confirming the successful incorporation of Ru and the consequent lattice expansion. These results are consistent with the observed shift in diffraction peaks to lower angles (Figure 1a).

The crystal structure of LSCR-0.1 was further examined by HRTEM. As shown in Figure 1b, two sets of clear lattice fringes corresponding to the perovskite phase are observed. The fringe with a spacing of 0.2682 nm can be assigned to the (104) plane of La_0.6_Sr_0.4_CoO_3_, which perfectly matches the standard PDF card (#36-1393). Another fringe with a spacing of 0.3752 nm corresponds to the (012) plane of La_0.6_Sr_0.4_CoO_3_. The clear presence and close combination of these lattice fringes not only confirm the integrity and high crystallinity of the perovskite crystal structure after Sr and Ru co-doping but also corroborate the XRD results showing no impurity phases and precisely matched characteristic peaks.

**Figure 2:**
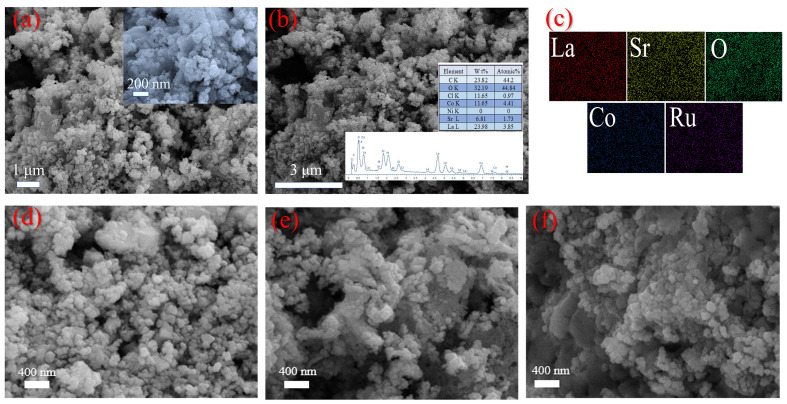
(**a**) SEM image of LSCR-0.1; (**b**,**c**) mapping image and elemental maps of La, Sr, O, Co, and Ru for LSCR-0.1; (**d**) SEM image of LSCR-0.05; (**e**) SEM image of LSC; and (**f**) SEM image of LSCR-0.15.

The SEM images in Figure 2a–f clearly reveal the differences in the microscopic morphology of the LSC, LSCR-0.05, LSCR-0.1, and LSCR-0.15 samples. The LSCR-0.1 sample (Figure 2a) displays a uniform sphere-like nanoscale morphology. Most particles possess sizes lying between 20 and 60 nm. Furthermore, the sample possesses a well-developed porous network and superior particle dispersibility, showing no obvious agglomeration. Although BET surface area measurements were not performed, the observed porous morphology is consistent with the increased electrochemically active surface area (ECSA) and enhanced OER kinetics presented in Section 3.3, supporting improved mass transport and active-site accessibility. Conversely, the micrographs reveal that LSCR-0.05 (Figure 2d) and pure LSC (Figure 2e) show extensive agglomeration and heterogeneous particle sizes. The over-doped LSCR-0.15 (Figure 2f) exhibits comparable aggregation issues, further exacerbated by a partial degradation of its porous framework. The low-magnification TEM image (Figure 1c) shows that LSCR-0.1 exhibits a uniform quasi-spherical nanoparticle morphology with a particle size distribution in the range of 20–60 nm, and a well-defined porous structure is formed between the particles. This is highly consistent with the excellent dispersion and porous features observed by SEM, which are beneficial for providing efficient mass transfer channels and abundant active sites for the electrocatalytic reaction.

The enhanced catalytic activity of LSCR-0.1 is driven by two critical factors. First, its uniform nanostructure and well-preserved porosity optimize mass transfer kinetics. Second, this structure maximizes the accessibility of its active sites. Complementing this, the EDS maps for LSCR-0.1 (Figure 2b,c) provide visual evidence of the uniform co-existence of La, Sr, Co, O, and Ru. The images show a complete absence of phase separation or localized clustering of any specific element. The measured Sr and Ru concentrations (0.4 and 0.1) are consistent with the designed stoichiometry, as confirmed by EDS. This finding underscores the effective lattice doping of Ru, resulting in a highly homogeneous solid-solution structure.

### 3.2. Characterization of Catalyst Elements and Valence States

**Figure 3:**
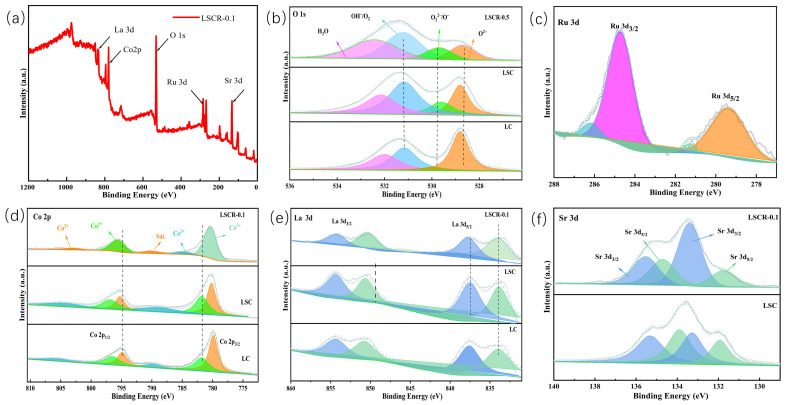
XPS characterization results: (**a**) Survey XPS spectrum of LSCR-0.1; (**b**) O 1s spectra of LC, LSC, and LSCR-0.1; (**c**) Ru 3d spectrum of LSCR-0.1; (**d**) Co 2p spectra of LC, LSC, and LSCR-0.1; (**e**) La 3d spectra of LC, LSC, and LSCR-0.1; (**f**) Sr 3d spectra of LSC and LSCR-0.1.

The surface composition and chemical valence states of the catalyst were analyzed by XPS. The survey spectrum (Figure 3a) confirms the presence of O, Ru, Co, La, and Sr elements in LSCR-0.1. In the high-resolution Sr 3d spectrum (Figure 3f), four peaks are observed. The doublets at 131.75/133.40 eV and 134.70/135.53 eV are assigned to Sr 3d_5/2_ and Sr 3d_3/2_, respectively. Regarding the Ru species, the high-resolution Ru 3d spectrum is deconvolved into four peaks. The peaks located at 279.36 and 284.71 eV (Ru 3d_5/2_ and 3d_3/2_) are attributed to Ru^4+^, confirming its dominance in the lattice. The presence of high-valence Ru^4+^ is expected to enhance the overall conductivity and electrocatalytic performance of the material. The electronic interaction between Ru and Co was further investigated by XPS. The presence of high-valence Ru^4+^ not only enhances electronic conductivity but also induces local lattice distortion, as evidenced by the XRD peak shifts, thereby facilitating the formation of oxygen vacancies and promoting the lattice oxygen participation in the OER. As shown in Figure 3d, the Co 2p spectrum of LSCR-0.1 exhibits a positive shift in binding energy compared to the pristine sample. This indicates a decreased electron density around the Co sites, suggesting that electrons are transferred from Co to the Ru dopants. This experimental observation aligns well with our DFT calculations, which reveal a charge redistribution from Co to the more electronegative Ru atoms, thereby effectively modulating the local electronic structure. Figure 3d illustrates the Co 2p region, resolved into two spin–orbit doublets (Co 2p_3/2_/Co 2p_1/2_) and two associated satellite peaks. The Co 2p spectrum of LSCR-0.1 features two main peaks at 780.36 eV and 795.6 eV. These are assigned to Co 2p_3/2_ and Co 2p_1/2_, respectively. Furthermore, each of the Co 2p_3/2_ and Co 2p_1/2_ peaks can be deconvolved into two distinct components, which can be assigned to Co^3+^ (780.4/795.5 eV) and Co^2+^ (783.72/802.48 eV). The binding energy of Co^2+^ exhibits a decrease in LSC and LC(LaCoO_3_) but an increase in LSCR-0.1. This trend is consistent with the shifts observed for the La 3d and Sr 3d peaks. The Co^3+^/(Co^2+^+ Co^3+^) ratios derived from the Co 2p spectra are 0.592 for LC, 0.623 for LSC, and 0.672 for LSCR-0.1, confirming the predominance of highly oxidative Co^3+^ species in LSCR-0.1. Thus, a higher oxidation state is observed in the Co 2p spectrum of LSCR-0.1.

As presented in Figure 3b, the O 1s spectra are fitted with four components: lattice O^2−^, highly oxidative O_2_^2−^/O^−^, surface -OH/O_2_, and adsorbed H_2_O. The positive shift in the O^2−^ binding energy upon Ru doping points to a more robust metal–oxygen bond configuration in LSCR-0.1. It has been reported that the abundance of O_2_^2−^/O^−^ species is related to the surface oxygen vacancy concentration [34]. The binding energies and relative proportions of the various oxygen species, determined by peak area fitting, are compiled in Table 1. With Ru doping, LSCR-0.1 exhibits a marked increase in highly oxidative O_2_^2−^/O^−^ species (from 12.95% to 21.35% relative to LSC), alongside a significant rise in -OH/O_2_ species. This increase in surface active oxygen species is consistent with the DFT-predicted upward shift in the O 2p-band center. The upshifted O 2p band implies stronger hybridization between metal 3d and O 2p orbitals, indicating increased covalency. The ratio of reactive oxygen species (O_2_^2−^/O^−^) increased significantly. This rise matches the DFT-predicted reduction in oxygen vacancy formation energy. Therefore, Ru doping effectively activated the lattice oxygen. Detailed XPS examinations confirm that charge redistribution across the Co–Ru interface in LSCR-0.1 generates abundant active sites (Co^2+^/Co^3+^, Ru^4+^ and O_2_^2−^/O^−^), thereby offering direct electronic evidence for its enhanced catalytic performance [35].

### 3.3. Characterization of the Catalyst’s Electrochemical Properties

The oxygen evolution reaction (OER) performance of the as-prepared catalysts was systematically assessed. Measurements were conducted in 1 M KOH electrolyte using a three-electrode system.

**Figure 4:**
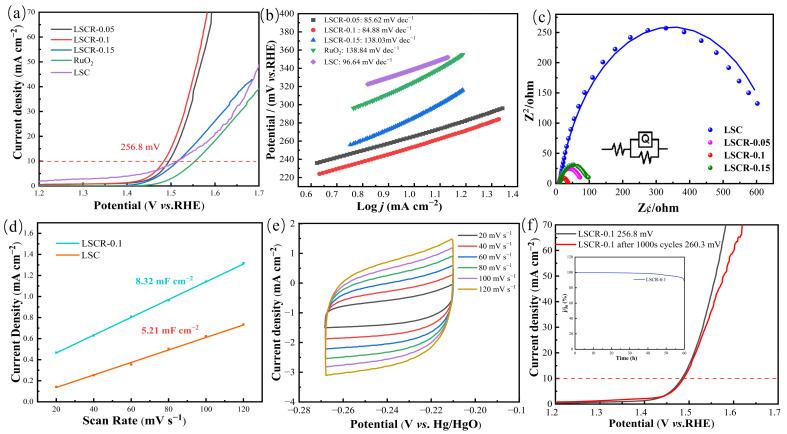
Electrochemical characterization results: (**a**) LSV curves and (**b**) Tafel slopes of LSC, LSCR-0.05, LSCR-0.1, LSCR-0.15, and RuO_2_; (**c**) EIS of LSC, LSCR-0.05, LSCR-0.1, and LSCR-0.15; (**d**) *C_dl_* of LSC and LSCR-0.1; (**e**) CV curves of LSCR-0.1 at scan rates from 20 to 120 mV/s; (**f**) electrocatalytic stability of LSC and LSCR-0.1 determined by different methods.

According to the LSV results in Figure 4a, the catalyst delivers a current density of 10 mA cm^−2^ at a low overpotential of 285.9 mV, 268.6 mV, 256.8 mV, 285.1 mV, and 330.2 mV for LSC, LSCR-0.05, LSCR-0.1, LSCR-0.15, and commercial RuO_2_, respectively. The superior OER activity of LSCR-0.1 over commercial RuO_2_ demonstrates that Ru doping effectively optimizes the catalyst’s electronic structure and surface active sites, leading to improved OER performance. As shown in Figure 4b, the Tafel slopes obtained from LSV curve fitting provide direct insight into the speed of electron transfer in the OER cycle. The Tafel slopes of LSC, LSCR-0.05, LSCR-0.1, LSCR-0.15, and RuO_2_ are 138.84 mV dec^−1^, 85.62 mV dec^−1^, 84.88 mV dec^−1^, 138.03 mV dec^−1^, and 96.64 mV dec^−1^, respectively. The smallest Tafel slope observed for LSCR-0.1 demonstrates its fastest OER kinetics, which facilitates more efficient charge transfer and transformation of reaction intermediates. The LSCR-0.1 catalyst integrates a lower reaction energy barrier with enhanced kinetics, resulting in superior overall OER performance, as evidenced by its low overpotential and Tafel slope. Compared with previously reported similar catalysts (Table S4), the LSCR-0.1 catalyst synthesized in this work exhibits superior oxygen evolution reaction (OER) performance, requiring an overpotential of only 256.8 mV at a current density of 10 mA cm^−2^. This significant performance enhancement is mainly attributed to the intrinsic structural advantages of the material, specifically including the synergistic effect of Sr and Ru in optimizing the electronic structure of LaCoO_3_, which has been confirmed by XPS and DFT calculations.

The ECSA of LSCR-0.1 was evaluated by CV measurements at 20–120 mV s^−1^ (Figure 4e). The Cdl value, obtained from the linear fit of current density against scan rate (Figure 4d), is 8.32 mF cm^−2^ for LSCR-0.1, which is higher than that of pristine LSC (5.21 mF cm^−2^). This indicates that Ru doping effectively increases the ECSA, exposing more active sites and providing sufficient reaction interfaces for OER. Furthermore, the intrinsic activity was evaluated by normalizing the current density to ECSA (see Table S5). LSCR-0.1 exhibits a substantially higher ECSA-normalized current density than LSC, confirming that the enhanced OER performance originates from both an increased number of active sites and superior intrinsic activity per site. EIS was applied to examine the interfacial charge transfer behavior of the catalysts.

The experimental Nyquist spectra and their corresponding equivalent circuit fittings are illustrated in Figure 4c. For a complete list of the fitted parameters, please refer to Table S1 in the Supplementary Materials. In the circuit, the solution resistance is labeled as *R_s_*, whereas the charge transfer resistance occurring at the electrode–electrolyte boundary is defined as *R_ct_*. LSCR-0.1 shows the smallest Nyquist semicircle and the lowest *R_ct_*, implying faster charge transfer kinetics. This finding is consistent with its excellent OER performance, demonstrating that Ru doping effectively enhances interfacial charge transport. The operational stability of the catalysts was assessed via chronoamperometry, as shown in Figure 4f. At a current density of 10 mA cm^−2^, the LSCR-0.1 catalyst exhibited excellent stability, with the normalized current density showing almost no decay over 40 h. The gradual decline observed after 40 h may be attributed to catalyst surface reconstruction, loss of active sites, or degradation of the electrode/electrolyte interface during prolonged OER operation. XRD analysis was conducted on the LSCR-0.1 catalyst after the 60 h stability test (Figure S1) [36,37,38,39,40,41,42,43,44]. The results show no significant changes in the crystal structure after testing, further confirming its structural and chemical integrity. After 1000 cycles of CV scanning, the LSV curve of LSCR-0.1 showed only a 3.5 mV negative shift compared with the initial curve. The observed data indicate that LSCR-0.1 possesses a strong capacity to withstand degradation under alkaline conditions, preserving its electrochemical efficiency and architectural coherence. This durability highlights the catalyst’s suitability for industrial applications.

### 3.4. DFT Analysis

The conventional OER typically follows the adsorbate evolution mechanism (AEM). In this mechanism, the rate-limiting step is controlled by a linear scaling relationship between the free energies of intermediate adsorption. As a result, the theoretical overpotential rarely exceeds 0.37 V.

**Figure 5:**
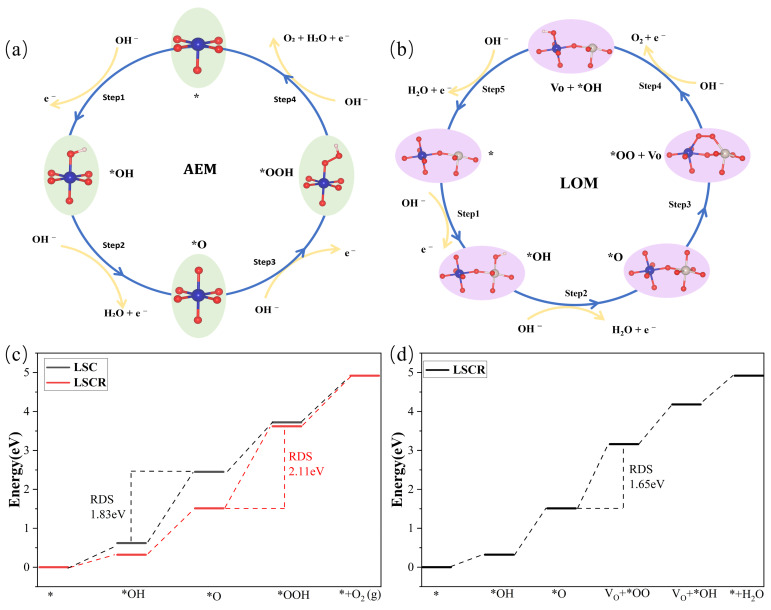
Schematic diagram of reaction pathways and Gibbs free energy plots. (**a**) Schematic diagram of the AEM pathway followed at the Co site in LSC. (**b**) Schematic diagram of the LOM pathway followed at the Ru site in LSCR. (**c**) Gibbs free energy diagram for LSC and LSCR along the AEM pathway. (**d**) Gibbs free energy diagram for LSCR along the LOM pathway, with the rate-determining step (RDS) indicated in the figure. Note: The asterisks (*) on the x-axis in (**c**,**d**) represent the catalysts in the reaction process.

In this work, DFT calculations reveal that Ru doping can shift the reaction pathway from AEM to the lattice oxygen oxidation (LOM) mechanism. This shift helps overcome the overpotential limitation. As shown in Figure 5a,c, the undoped LSC exhibits a rate-determining step (RDS) at the Co site: *OH → *O. The Gibbs free energy change for this step is ΔG = 1.83 eV. The corresponding Gibbs free energy changes (ΔG) for each elementary step are provided in Table S6 in the Supplementary Materials. After Ru doping, the Ru–O bond becomes significantly strengthened (see COHP analysis). Key intermediate structures for LSC (AEM, Figure S2) and LSCR-0.1 (AEM/LOM, Figure S3) are in the Supplementary Materials.

This leads to the over-stabilization of the O intermediate. Consequently, the energy barrier for the subsequent step (O → OOH) increases to 2.11 eV. Thus, the AEM pathway becomes thermodynamically unfavorable. In contrast, when considering the LOM pathway (Figure 5b–d), the O intermediate can directly couple with a neighboring lattice oxygen. This forms OO and generates an oxygen vacancy (V_o_). The RDS energy barrier for this process drops to 1.65 eV. The corresponding theoretical overpotential decreases significantly, from 0.88 V (AEM) to 0.43 V (LOM). This result is consistent with experimental observations. The OER overpotential of LSCR-0.1 (256.8 mV) is much lower than that of LSC (285.9 mV) and commercial RuO_2_ (330.2 mV). LSCR-0.1 also shows the lowest Tafel slope (84.88 mV dec^−1^), indicating faster reaction kinetics. This is a direct manifestation of the LOM pathway bypassing the OOH formation bottleneck, thereby achieving highly efficient O–O coupling.

**Figure 6:**
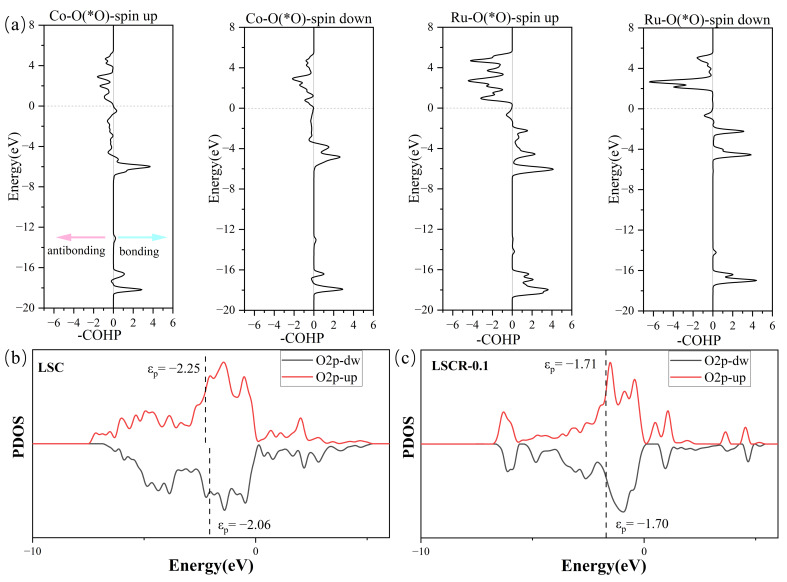
(**a**) COHP analysis of Co-O(O) and Ru-O(O); the left panel shows the anti-bonding state, and the right panel shows the bonding state. (**b**) O 2p PDOS for lattice oxygen in the LSC. (**c**) O 2p PDOS for lattice oxygen in the LSCR.

To understand why Ru doping triggers the LOM pathway, we analyzed the electronic structure using the crystal orbital Hamiltonian population (COHP) and the projected density of states (PDOS). The COHP results (Figure 6a) show that, below the Fermi level, the Ru–O(O) bond has more bonding states than the Co–O(O) bond. At the same time, its anti-bonding states are greatly reduced. This confirms a stronger covalent interaction between Ru and the adsorbed oxygen. As a direct result, the adsorption free energy of *O dropped significantly, from 2.45 eV to 1.51 eV. More importantly, the PDOS analysis (Figure 6b,c) reveals that Ru doping shifts the p-band center of the lattice oxygen (O 2p orbital). It moves from −2.25 eV to −1.71 eV, closer to the Fermi level. An upward shift in the p-band center means higher electronic activity of lattice oxygen and stronger M–O covalency. This not only promotes oxygen vacancy formation but also helps lattice oxygen participate directly in O–O bond formation. This electronic feature agrees well with experimental XPS results. In LSCR-0.1, the proportion of high-oxidation-state oxygen species (O_2_^2−^/O^−^) increased from 12.95% to 21.35% (Table 1). This indicates a higher concentration of surface oxygen vacancies, which provides the structural basis for the LOM pathway. In summary, Ru doping modulates metal–oxygen electron coupling. It simultaneously optimizes the oxidation state of active sites (increasing Co^3+^ proportion), lattice oxygen activity, and oxygen vacancy concentration. Together, these factors enable a shift from the AEM to the LOM pathway, leading to a significant improvement in OER performance. Conclusive confirmation of the LOM pathway requires direct experimental evidence, such as in situ Raman spectroscopy combined with ^18^O isotope labeling [45,46]. However, our indirect evidence—an upshifted O 2p-band center, reduced oxygen vacancy formation energy, and an increased proportion of O_2_^2−^/O^−^ species—collectively points to lattice oxygen activation. The consistency between DFT, XPS, and electrochemical data indicates that Ru doping triggers the transition from AEM to LOM, which agrees well with conclusions from operando and isotope-labeling studies on related perovskite systems reported in the literature [47]. A recent review also recognizes the value of combining multiple indirect lines of evidence [48]. Future work will employ in situ/operando techniques to directly validate this mechanism. Computation methods are in the Supplementary Materials [49,50,51,52,53,54].

## 4. Conclusions

In summary, this work successfully combines DFT calculations with experimental validation to elucidate how the combination of A-site substitution and B-site doping regulates the OER performance of La_0.6_Sr_0.4_Co_0.9_Ru_0.1_O_3_ perovskites.

Theoretical calculations show that Ru doping induces a transition from the conventional AEM to the more efficient LOM pathway. This is attributed to the enhanced adsorption of *O intermediates and the significantly reduced formation energy of oxygen vacancies, which are rooted in the upward shift in the O 2p-band center.

Experimentally, the sol–gel synthesized LSCR-0.1 catalyst confirms these predictions, exhibiting optimized electronic conductivity and a high density of active sites. The catalyst demonstrates a low overpotential (256.8 mV @ 10 mA cm^−2^), a small Tafel slope (84.88 mV dec^−1^), and robust stability over 10 h, outperforming the benchmark RuO_2_ catalyst.

This study confirms that the synergistic regulation of lattice structure and electronic states via A/B co-modulation is a highly effective strategy for developing advanced non-noble metal OER electrocatalysts.

## Figures and Tables

**Table 1:** Relative concentrations of different oxygen species derived from O 1s XPS analysis.

Perovskite Catalysts		O 1s				Area Ratio O_2_^2−^/O^−^
		O^2−^	O_2_^2−^/O^−^	OH^−^/O_2_	H_2_O	O^2−^ + OH^−^/O_2_ + H_2_O
LSC	Position [eV]	528.81	529.60	531.17	532.15	0.149
	Proportion [%]	21.55	12.95	40.39	25.11	
LSCR-0.1	Position [eV]	528.90	531.86	531.26	532.42	0.277
	Proportion [%]	23.23	21.35	32.25	23.17	

## Data Availability

The original contributions presented in this study are included in the article/Supplementary Materials. Further inquiries can be directed to the corresponding author.

## Supplementary Materials

The following supporting information can be downloaded at https://www.mdpi.com/article/10.3390/ma19112383/s1. Figure S1: XRD patterns of the LSCR-0.1 catalyst before and after the 60 h chronoamperometry stability test at 10 mA cm^−2^ in 1 M KOH; Table S1: Fitting of OER EIS parameters for catalyst samples in alkaline electrolytes; Figure S2: Structures of the key intermediates on the AEM pathway of LSC; Figure S3: Structures of the key intermediates on the AEM and LOM pathway of LSCR-0.1; Table S2: Crystallite sizes calculated from the perovskite (104) diffraction peak using the Scherrer equation; Table S3: Rietveld refinement results of lattice parameters for LSC and LSCR-x samples; Table S4: OER activities of this work at 10 mA cm^−2^ were compared with those of previously reported similar catalysts in 1 M KOH solution; Table S5: ECSA values of LSC and LSCR-0.1 samples in alkaline electrolyte; Table S6: Gibbs free energy changes (ΔG) for each elementary step of the AEM and LOM pathways. References [35,36,37,38,39,40,41,42,43,44,49,50,51,52,53,54] are cited in the Supplementary Materials.
