# Supplementary material for: B-Site Ru Doping in Sr-Substituted LaCoO3 Perovskite for Enhanced OER Performance: A Combined Experimental and DFT Study
Source: Materials (Basel). 2026 Jun 3;19(11):2383. doi: 10.3390/ma19112383 (PMC13258156; doi:10.3390/ma19112383)
Supplement: Supplementary file 1 [file materials-19-02383-s001.zip › materials-4309153-supplementary.pdf]

B-Site Ru Doping in Sr-Substituted LaCoO3 Perovskite for Enhanced OER Performance: A Combined Experimental and DFT Study

Lina Zhang, Tian Fang, Changhai Liu, Wenchang Wang, Shiying Wang and Zhidong Chen *

School of Materials Science and Engineering, Changzhou University, Changzhou 213164, China

* Correspondence：zdchen@cczu.edu.cn

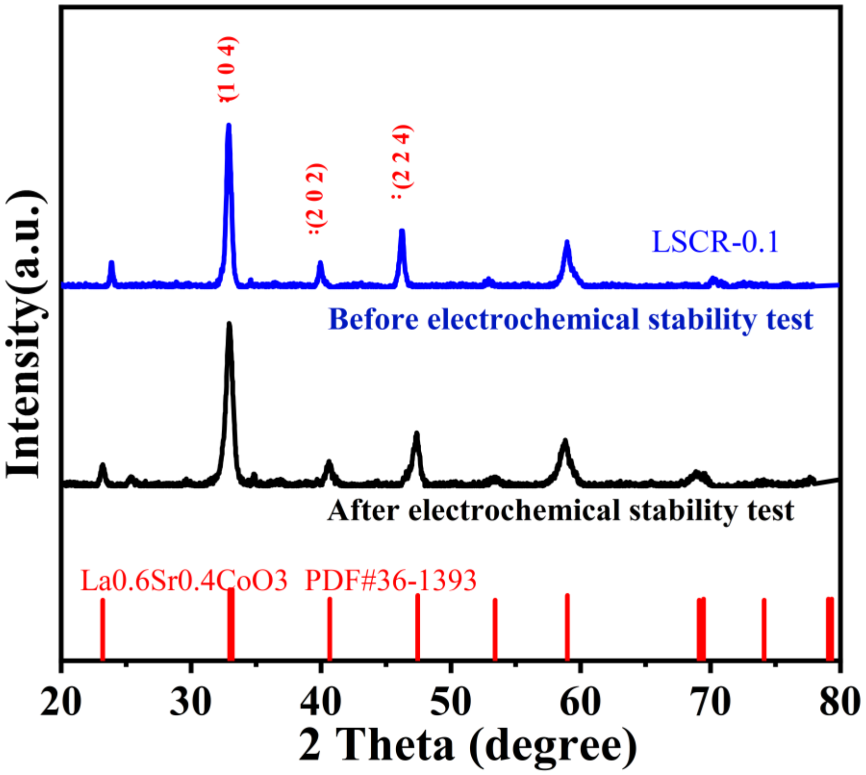

Figure S1. XRD patterns of the LSCR-0.1 catalyst before and after the 60 h chronoamperometry stability test at 10 mA cm^{-2} in 1 M KOH.

Table S1. Fitting of OER EIS parameters for catalyst samples in alkaline electrolytes

Catalyst	R_t (Ω)/Error(%)	Q/Error(%)	n/Error(%)	R_{CT} (Ω)/Error(%)
LSC	10.62, 2.416	0.0006496, 5.465	0.8369, 1.592	671.4, 5.290
LSCR-0.05	14.21, 2.507	0.0011250, 5.655	0.8157, 1.564	89.5, 6.562
LSCR-0.1	31.31, 2.114	0.0045691, 12.28	0.6452, 6.546	31.27, 7.523
LSCR-0.15	14.56, 1.352	0.0036450, 9.245	0.8455, 4.456	111.34, 2.546

Computation methods

DFT calculations were performed by using the Vienna Ab-initio Simulation Package (VASP)[1,2]. The exchange–correlation interactions were described by generalized gradient approximation (GGA)[3] with the Perdew–Burke–Ernzerhof (PBE) functional[4]. Spin-polarization was included in all the calculations, and a damped van der Waals correction was incorporated using Grimme's scheme to better describe the non-bonding interactions[5]. A plane wave cut-off energy of 500 eV was used, and 3×3×1 Monkhorst–Pack grid k-points were employed. The residual force and energy on each atom during structure relaxation were converged to 0.005 eV $Å^{-1}$ and 10^{-5} eV, respectively. The Hubbard model (DFT+U) was utilized, where the effective U (Ueff) values were set to 4.0 eV for Co and 3.0 eV for Ru, respectively [6].

In the OER, the AEM step and the corresponding Gibbs free energy change can be expressed as follows:

$$* + OH^- \rightarrow *OH + e^- \quad (S1)$$

$$\Delta G_1 = G_{(*OH)} + G_{(H_2)}/2 - G_{(*)} - G_{(H_2O)} \quad (S2)$$

$$*OH + OH^- \rightarrow *O + H_2O + e^- \quad (S3)$$

$$\Delta G_2 = G_{(*O)} + G_{(H_2)}/2 - G_{(*OH)} \quad (S4)$$

$$*O + OH^- \rightarrow *OOH + e^- \quad (S5)$$

$$\Delta G_3 = G_{(*OOH)} + G_{(H_2)}/2 - G_{(*O)} - G_{(H_2O)} \quad (S6)$$

$$*OOH + OH^- \rightarrow * + O_2 + H_2O + e^- \quad (S7)$$

$$\Delta G_4 = G\_{(O_2)} + G_{(*)} + G_{(H_2)}/2 - G_{(*OOH)} \quad (S8)$$

The LOM step and the corresponding Gibbs free energy change can be expressed as follows:

$$* + OH^- \rightarrow *OH + e^- \quad (S9)$$

$$\Delta G_1 = G_{(*OH)} + G_{(H_2)}/2 - G_{(*)} - G_{(H_2O)} \quad (S10)$$

$$*OH + OH^- \rightarrow *O + H_2O + e^- \quad (S11)$$

$$\Delta G_2 = G_{(*O)} + G_{(H_2)/2} - G_{(*OH)} \quad (S12)$$

$$*O \rightarrow *(V_O)O_2 \quad (S13)$$

$$\Delta G_3 = G_{(*(V\_O)O_2)} - G_{(*O)} \quad (S14)$$

$$*(V_O)O_2 + OH^- \rightarrow *(V_O)OH + O_2 + e^- \quad (S15)$$

$$\Delta G_4 = G_{(*(Vo)OH)} + G_{(O_2)} + G_{(H_2)}/2 - G_{(*(Vo)O_2)} - G_{(H_2O)} \quad (S16)$$

$$*(V_O)OH + OH^- \rightarrow * + H_2O + e^- \quad (S17)$$

$$\Delta G_5 = G\_{(*)} + G\_(H_2)/2 - G_{(*(Vo)OH)} \quad (S18)$$

The Gibbs free energy of adsorption is adjusted by the zero-point energy (ΔZPE) and entropy (ΔS):

$$\Delta G = \Delta E + \Delta ZPE + T\Delta S \quad (S19)$$

The energy difference between the reactants and products in a chemical reaction is represented as ΔE, which includes the zero-point energy adjustment (ΔZPE). Furthermore, the variation in

vibrational entropy at a given temperature T is indicated as ΔS.

To evaluate the performance of OER, the overpotential η_{OER} could be calculated using the following method:

$$\eta_{OER} = \max(\Delta G_1, \Delta G_2, \Delta G_3, \Delta G_4)/e - 1.23 \quad (S20)$$

Here, max(ΔG_1, ΔG_2, ΔG_3, ΔG_4) refers to the step with the highest energy release among the four steps, which corresponds to the thermodynamic rate-determining step (RDS).

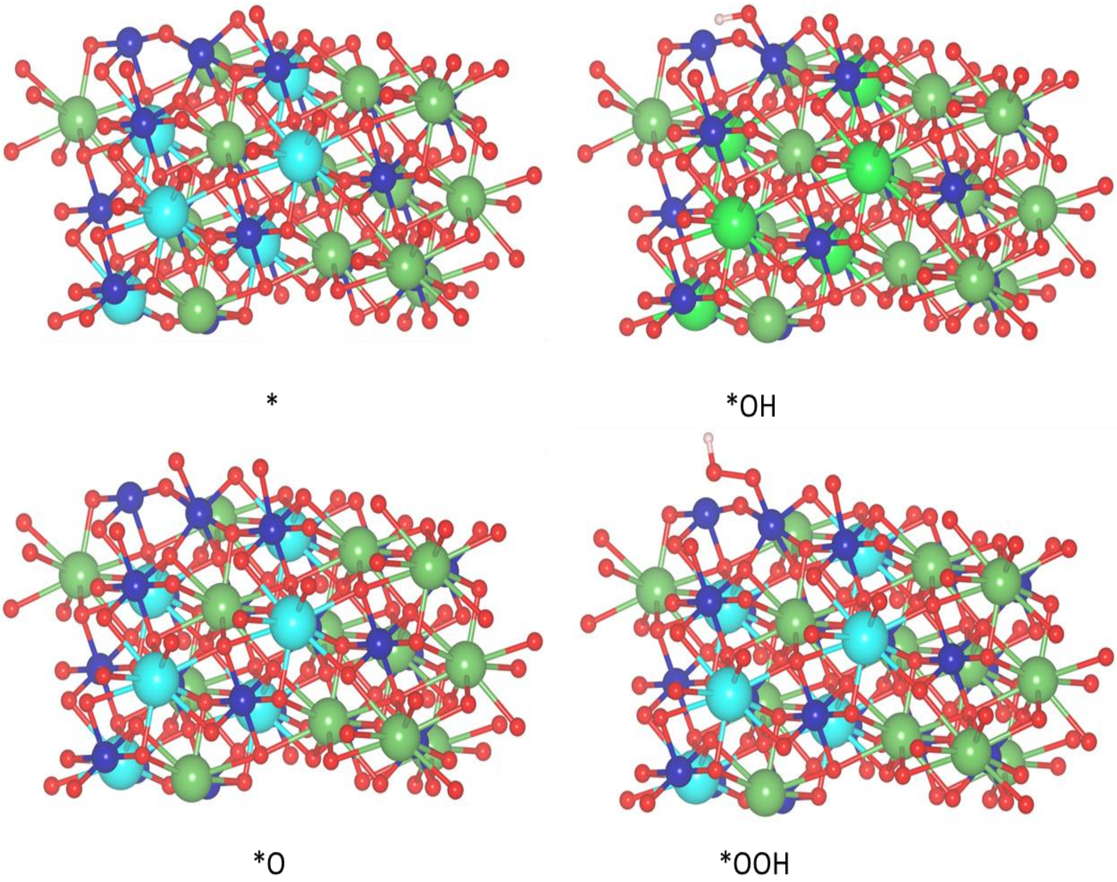

Figure S2. Structures of the key intermediates on the AEM pathway of LSC.

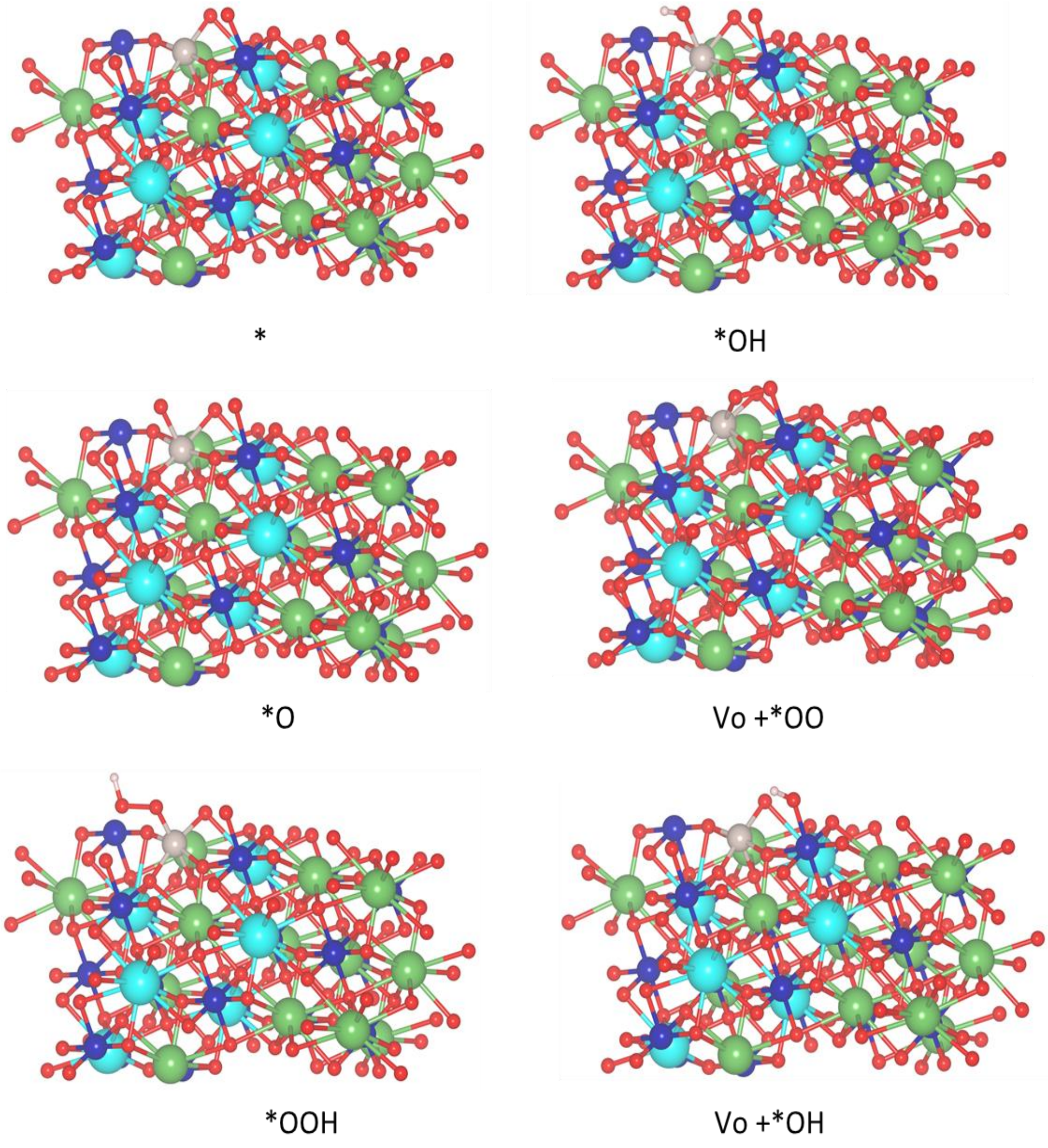

Figure S3. Structures of the key intermediates on the AEM and LOM pathway of LSCR-0.1.

Table S2. Crystallite sizes calculated from the perovskite (104) diffraction peak using the Scherrer equation.

Sample	2-Theta	FWHM (°)	Crystallite size (nm)
LSC	32.90	0.516	17.84
LSCR0.05	32.86	0.469	19.62
LSCR0.1	32.80	0.506	18.18
LSCR0.15	32.71	0.583	15.78

Table S3. Rietveld refinement results of lattice parameters for LSC and LSCR-x samples.

Sample	a (Å)	b (Å)	c (Å)	R_{wp} (%)	GOF (χ^2)
LSC	5.43756	5.43756	13.1437	4.23	1.15
LSCR-0.05	5.43882	5.43882	13.19753	4.56	1.21
LSCR-0.1	5.44546	5.44546	13.20932	4.97	1.28
LSCR-0.15	5.4654	5.4654	13.24568	5.14	1.34

NOTE：Space group: R-3c, R_{wp}, and GOF are defined in the Rietveld refinement.

Table S4. OER activities of this work at 10 mA cm^{-2} were compared with those of previously reported similar catalysts in 1 M KOH solution

Catalyst	Overpotential (mV) (mV vs. RHE)	Tafel slope (mV dec^{-1})	Electrode	Ref.
**LSCR-0.1**	**256.8**	**84.88**	**Glassy carbon**	**This work**
S-doped $LaCoO_3$	364	126.7	Glassy carbon	[7]
$LaCo_{0.94}Pt_{0.06}O_{3-\delta}$	454	86	Glassy carbon	[8]
$La_{0.96}Ce_{0.04}CoO_3$	380	80	Glassy carbon	[9]
$LaCo_{0.8}Ru_{0.2}O_3$	460	43	Glassy carbon	[10]
$SrCo_{0.9}Ru_{0.1}O_3$	360	113	Glassy carbon	[11]
$La_{1-x}Ce_xNiO_3$	270	45	Carbon paper	[12]
$La_{0.9}Ce_{0.}1CoO_3$	343	64	Nickel foam	[13]
$La_{0.9}Ca_{0.1}CoO_3$	385	83	Nickel foam	[13]
$La_{0.7}Ba_{0.3}CoO_3$	402	98	Nickel foam	[13]
$La_{0.7}Sr_{0.3}CoO_{3-\delta}$	326	70.8	Glassy carbon	[14]
Sr_2CoFeO_6	300	59.62	Glassy carbon	[15]
$La_{0.5}Sr_{0.5}Co_{0.75}Fe_{0.25}O_3$	310	107.03	carbon cloth	[16]

Table S5. ECSA values of LSC and LSCR-0.1 samples in alkaline electrolyte.

	C (F/g)	ECSA (m^2/g)
LSC	7.99	13.29
LSCR-0.1	12.76	21.27

Table S6. Gibbs free energy changes (ΔG) for each elementary step of the AEM and LOM pathways.

	LSC-AEM	LSCR-AEM	LSCR-LOM
ΔG_1	0.62 eV	0.32 eV	0.32 eV
ΔG_2	1.83 eV	1.19 eV	1.19 eV
ΔG_3	1.26 eV	2.11 eV	1.65 eV
ΔG_4	1.21 eV	1.30 eV	1.02 eV
ΔG_5	/	/	0.74 eV

References

[1] Kresse G, Furthmüller J, et al. Furthmüller b.Efficiency of ab-initio total energy calculations for metals and semiconductors using a plane-wave basis set-ScienceDirect[J]. Computational Materials Science, 1996, 6(1):15-50.

[2] Kresse G, Furthmüller J. Efficient Iterative Schemes for Ab Initio Total-Energy Calculations Using a Plane-Wave Basis Set[J]. Physical review. B, Condensed matter, 1996, 54:11169.

[3] Perdew JP, Burke K, Ernzerhof M. Generalized Gradient Approximation Made Simple[J]. Physical review letters, 1996, 77: 3865-3868.

[4] Perdew JP, Ernzerhof M, Burke K. Rationale for mixing exact exchange with density functional approximations[J]. Journal of Chemical Physics, 1996, 105(22):9982-9985.

[5] Grimme S. Semiempirical GGA-type density functional constructed with a long-range dispersion correction[J]. Journal of Computational Chemistry, 2006, 27(15):1787-1799.

[6] Kong FT, Longo RC, Park MS, Yoon J, Yeon DH, Park JH, Wang WH, Santosh KC, Doo SG, Cho K. Ab initio study of doping effects on $LiMnO_2$ and Li_2MnO_3 cathode materials for Li-ion batteries[J]. Journal of Materials Chemistry A, 2015, 3(16): 8489-8500.

[7] Ran J, Wang T, Zhang J, et al. Modulation of Electronics of Oxide Perovskites by Sulfur Doping for Electrocatalysis in Rechargeable Zn–Air Batteries[J]. Chemistry of Materials, 2020, 32: 3439–3446.

[8] Wang C, Zeng L, Guo W, et al. Enhancing oxygen and hydrogen evolution activities of perovskite oxide $LaCoO_3$ via effective doping of platinum[J]. Royal Society of Chemistry Advances, 2019, 9(61):35646-35654.

[9] Li WY, Zhu YM, Guo W, et al. Enhanced oxygen and hydrogen evolution activities of Pt/$LaCoO_3$ perovskite oxide via in-situ exsolution of Pt nanoparticles[J]. Journal of Chemical Sciences, 2022, 134: 38.

[10] Chandrappa SG, Moni P, Chen D, et al. The influence of ruthenium substitution in $LaCoO_3$ towards bi-functional electrocatalytic activity for rechargeable Zn–air batteries[J]. Journal of Materials Chemistry A, 2020, 8: 20612-20620.

[11] Dai J, Zhu YL, Yin YC, et al. Super-exchange interaction induced overall optimization in ferromagnetic perovskite oxides enables ultrafast water oxidation[J]. Small, 2019, 15: 1903120.

[12] Sun Y, Li R, Chen X, et al. A-Site Management Prompts the Dynamic Reconstructed Active Phase of Perovskite Oxide OER Catalysts[J]. Advanced Energy Materials, 2021, 11: 2003755.

[13] Hou XY, Li JQ, Shi C, et al. Nanoparticles of $LaCoO_3$ Doped with Ce, Sr, Ca, Ba on A-Site as Catalysts for the Alkaline Oxygen Evolution Reaction[J]. ACS Applied Nano Materials, 2025, 8(41): 19901-19909.

[14] Lu Y, Ma A, Yu YF, Tan R, Liu CW, Zhang P, Liu D, Gui JZ. Engineering Oxygen Vacancies into LaCoO3 Perovskite for Efficient Electrocatalytic Oxygen Evolution[J]. ACS Sustainable Chem. Eng. 2019, 7: 2906−2910.

[15] Li SF, Zheng J, Hu L, Ma Y, Yan D. Facile surface defect engineering on perovskite oxides for enhanced OER performance[J]. Dalton transactions: An international journal of inorganic chemistry,2023, 52(13): 4207-4213.

[16] Ge B, Jiang P, Chen B, Huang C. Controlling Co 3d/O 2p orbital hybridization in $LaCoO_3$ by modulating the Co–O–Co bond angle for enhanced oxygen evolution reaction catalysis. ACS Catalysis, 2025, 15(1): 477-486.
